# Stability of Methylphenidate under Various pH Conditions in the Presence or Absence of Gut Microbiota

**DOI:** 10.3390/ph14080733

**Published:** 2021-07-27

**Authors:** Julia Aresti-Sanz, Markus Schwalbe, Rob Rodrigues Pereira, Hjalmar Permentier, Sahar El Aidy

**Affiliations:** 1 Host-Microbe Interactions, Groningen Biomolecular Sciences and Biotechnology Institute (GBB), University of Groningen, 9747 AG Groningen, The Netherlands; j.aresti.sanz@rug.nl (J.A.-S.); m.schwalbe@rug.nl (M.S.); 2 Medical Center Kinderplein, 3083 BB Rotterdam, The Netherlands; robpereirar@gmail.com; 3 Interfaculty Mass Spectrometry Center, Department of Analytical Biochemistry, Groningen Research Institute of Pharmacy (GRIP), 9713 AV Groningen, The Netherlands; h.p.permentier@rug.nl

**Keywords:** Retalin, metabolism, availability, intestinal pH, gut bacteria

## Abstract

Methylphenidate is one of the most widely used oral treatments for attention-deficit/hyperactivity disorder (ADHD). The drug is mainly absorbed in the small intestine and has low bioavailability. Accordingly, a high interindividual variability in terms of response to the treatment is known among ADHD patients treated with methylphenidate. Nonetheless, very little is known about the factors that influence the drug’s absorption and bioavailability. Gut microbiota has been shown to reduce the bioavailability of a wide variety of orally administered drugs. Here, we tested the ability of small intestinal bacteria to metabolize methylphenidate. In silico analysis identified several small intestinal bacteria to harbor homologues of the human carboxylesterase 1 enzyme responsible for the hydrolysis of methylphenidate in the liver into the inactive form, ritalinic acid. Despite our initial results hinting towards possible bacterial hydrolysis of the drug, up to 60% of methylphenidate is spontaneously hydrolyzed in the absence of bacteria and this hydrolysis is pH-dependent. Overall, our results indicate that the stability of methylphenidate is compromised under certain pH conditions in the presence or absence of gut microbiota.

## 1. Introduction

Attention-deficit/hyperactivity disorder (ADHD) is one of the most prevalent neurodevelopmental disorders, affecting 6–12% of children and persisting into adulthood in around 60% of the cases [1]. Although a cause–effect relationship has not yet been established for ADHD, altered levels of dopamine and norepinephrine, and their corresponding transporters in the brain, seem to play a key role in the cognitive impairment and dysregulated reward system that characterize ADHD [2,3]. Thus, ADHD is mainly treated with amphetamine-like psychostimulants that improve symptoms by increasing the levels of dopamine and norepinephrine neurotransmitters in the brain.

Methylphenidate (MPH), a dopamine reuptake inhibitor, is considered as the gold-standard treatment for ADHD [4,5]. MPH is administered orally and rapidly absorbed into the bloodstream through the small intestine (SI), reaching peak concentrations between 1 and 3 h post-ingestion. Around 70% of MPH is recovered in urine in the form of ritalinic acid (RA). RA is the inactive metabolite of MPH produced in the liver by carboxylesterase 1 (CES1) [6]. Despite its efficacy, MPH has a low bioavailability of around 30%. Moreover, there is a high interindividual variability among patients in terms of their response to the treatment [7,8].

First-pass metabolism could explain the low bioavailability of MPH. Genetic variations in CES1, the gene encoding for the CES1 enzyme in the liver, have been shown to impact the enzyme activity towards different substrates in vitro [9], which could account for differences in MPH hydrolysis among patients. Nevertheless, very little is known about the stability of the drug and whether this could contribute to its low bioavailability in the gut [10,11]. Only recently, the absorption of MPH was modelled based on physicochemical properties of the drug, formulation-related information, and differences in gut physiology along the gastrointestinal tract [12]. Importantly, non-specific intestinal loss of MPH had to be introduced in the model in order to obtain plasma profiles of MPH and RA comparable to those found in clinical studies. Thus, the model suggested intestinal loss of MPH prior to its absorption and hepatic/systemic metabolism [12]. Nonetheless, the factors underlying the predicted non-specific intestinal loss remain to be explained.

The gut microbiota represents a metabolic factory able to metabolize indigenous and exogenous compounds, such as food components and drugs, in the host [13]. Direct bacterial metabolization of MPH could therefore explain the potential intestinal loss of MPH. Indeed, bacterial esterases that can hydrolyze carboxyl esters have been previously described [14]. For example, the highly abundant gut bacterium *Escherichia coli* harbors the esterase yjfP, which was shown to hydrolyze the ester 4-nitrophenylacetate [15]. Similarly, *Bacillus subtilis* pnbA esterase has been shown to hydrolyze 4-nitrophenylacetate [16]. Another plausible indirect effect of gut microbiota on the MPH bioavailability is through bacterial products of their metabolization of food components, which in turn, causes changes in the gut luminal pH [17]. Gut luminal pH is a key factor in regulating the absorption of drugs through the gut epithelium [18]. Analogously, the diet composition is another important contributor to pH changes in the small intestine [19]. The pharmacokinetic properties of MPH have been previously described [20]. However, to the best of our knowledge, there is a surprising lack of information about factors, such as pH and dietary components, that might affect the stability and intestinal absorption of such a well-known drug as MPH. Considering that hydrolysis is one of the main chemical reactions by which gut bacteria can metabolize drugs [21] and that esters such as MPH are susceptible to non-enzymatic pH-dependent hydrolysis [22], we sought to investigate the possible effect of these factors on the MPH stability in vitro.

## 2. Results

### 2.1. Gut Bacteria Harbor Homologues for the Human CES1 Enzyme Responsible for the Metabolization of Methylphenidate

**Figure 1:**
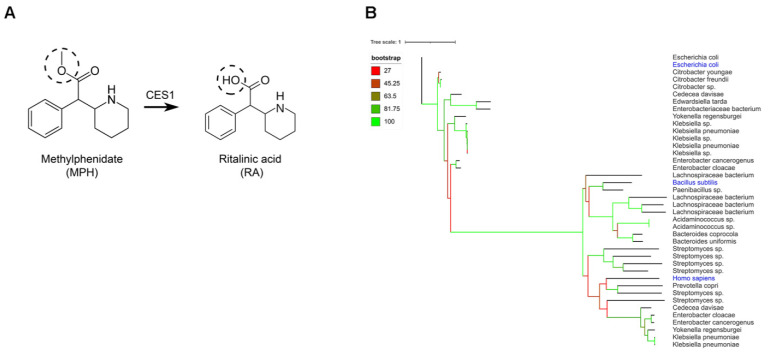
In silico analysis of bacterial homologues of the human carboxylesterase 1. (**A**) Hydrolysis reaction by human carboxylesterase 1 (CES1), which removes a methyl group from methylphenidate (MPH) to form ritalinic acid (RA). (**B**) Phylogenetic trees created using iTOL online tool showing gut bacterial strains harboring homologue enzymes of human CES1, *E. coli* yjfP and *B. subtilis* pnbA, respectively.

Carboxylesterase 1 (CES1) is the human enzyme responsible for the inactivation of MPH by hydrolysis of the ester group to form RA (Figure 1A) in the liver [6]. We examined whether the gut bacteria may harbor a homologue for the human CES1 enzyme. To this end, the protein sequence (XP_005255831.1) from the human CES1 enzyme was used as a query to search the US National Center of Health Human Microbiome Project (HMP) protein database. Additionally, we included in our search the protein sequence (ANK_04958.1) from *E. coli* yjfP esterase, as well as the protein sequence of *B. subtilis* pnbA (KIX_83209.1). The analysis revealed 30 closest orthologues with an identity higher than 30% and 90% query cover. (Figure 1B). Among the top identified bacteria were *E. coli* strains, which are highly abundant in the small intestine [23]. However, most of the identified bacteria carrying esterase orthologues were either pathogenic (*Klebsiella* sp.) or anaerobic bacteria (e.g., *Lachnospiraceae bacterium*, *Bacteroides uniformis*), which are not representative of the small intestinal microbiota. In order to broaden our follow-up screening of live small intestinal bacteria, we also searched in the unfiltered data from the yjfP orthologues analysis, to include predicted bacterial esterases below the 30% identity cut-off. This revealed more small intestinal bacteria, including *Enterococcus faecalis*, *Enterococcus faecium*, *Lactobacillus plantarum* and *Lactobacillus salivarius* (Figure S1, Supplementary Materials), harboring an esterase gene, which are abundant in the small intestine [24]. Our in-silico analysis confirms the presence of esterase enzymes that can hydrolyze MPH among gut microbiota in the small intestine, the main site of MPH absorption [6].

### 2.2. Conversion of Methylphenidate into Ritalinic Acid in Complex and Pure Bacterial Culture

**Figure 2:**
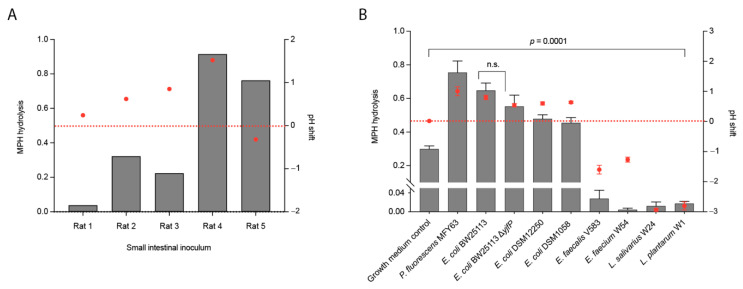
Methylphenidate hydrolysis in complex and pure bacterial cultures. (**A**) Methylphenidate hydrolysis by small intestinal luminal microbiota from WTG rats (*n* = 5) (grey bars; left *y*-axis) and pH changes during incubation with 50 µM methylphenidate at pH 7.0 (red dots; right *y*-axis). (**B**) Methylphenidate hydrolysis by gut bacterial pure cultures (grey bars; left *y*-axis) and pH changes during incubation with 50 µM methylphenidate at pH 7.0 (red dots; right *y*-axis). Hydrolysis is shown as the ratio of [RA]_24h_/[MPH]_0h_ quantified in ng/µL and normalized to d10-ritalinic acid. pH changes during bacterial growth are shown as pH shift after 24 h. Error bars represent standard deviation (S.D.) (*n* = 3).

To determine whether the small intestinal complex bacterial community can indeed metabolize and inactivate MPH prior to its absorption, small intestinal luminal samples from wild-type Groningen rats (*n* = 5) were incubated aerobically in vitro with 50 µM MPH in an enriched beef broth based on SHIME medium [25] (Table S1, Supplementary Materials). The concentration of MPH employed was based on the proposed estimation that well-absorbed drugs are present in the small intestine at concentrations ≥20 µM [26]. Concentrations of MPH and the hydrolysis product, RA, were monitored by high-performance liquid chromatography coupled with tandem mass spectrometry (HPLC-MS/MS). Analytical details for the quantification method of both analytes are provided in Figure S2. Interestingly, there was a wide variation among the tested luminal samples in their ability to convert MPH into RA, ranging from samples that metabolized 90% of MPH to RA, to samples where MPH was not metabolized to RA at all after 24 h (Figure 2A). The results suggest that small intestinal microbiota might be involved in the hydrolysis of the MPH. Importantly, only the starting pH (pH 7.0) as well as the pH shift at the end of the experiment (Figure 2A, Table 1) were monitored. The final pH differences among the tested samples did not correlate with the percentage of metabolized MPH (r = 0.22; r^2^ = 0.05; *p* value = 0.7153). This does not exclude the possibility that the temporary pH shifts during the course of the experiment due to the dynamic changes in bacterial growth and metabolic products was the cause of the MPH hydrolysis observed. However, it was not possible in our experimental setup to monitor the pH shift during the course of the experiment. 

In order to better decipher the role of bacterial hydrolysis on the MPH stability, pure bacterial cultures were employed. Based on our in-silico analysis, a comprehensive screening of gut-associated bacterial strains harboring esterase proteins was performed. We focused on the gut bacteria known to inhabit the small intestine, the major site of MPH absorption [12]. To this end, pure cultures of the Gram-negative bacteria *Pseudomonas fluorescens* MFY63, *Escherichia coli* DSM12250, *E. coli* DSM1058, and the laboratory strain *E. coli* BW25113 were incubated aerobically with 50 µM of MPH. Conversion of MPH to RA was analyzed by HPLC-MS/MS (details of the quantification method in these cultures are shown in Figure S2). *P. fluorescens* MFY63 and *E. coli* BW25113 cultures displayed a conversion of 70% of MPH into RA after 24 h of aerobic incubation (Figure 2B). In case of the gut isolates *E. coli* DSM1058 and *E. coli* DSM12250, 50% of MPH was hydrolyzed. In contrast to the hydrolysis observed when MPH was incubated with Gram-negative bacteria, the Gram-positive bacteria *E. faecalis* V583, *E. faecium* W54, *L. plantarum* W1, *L. salivarius* W24 and *C.* *ammoniagenes* DSM20306 cultures did not metabolize MPH (Figure 2B). Collectively, the results suggest that only Gram-negative bacteria harboring the esterase enzyme are involved in the metabolism of MPH. However, we noted that around 20% MPH was spontaneously hydrolyzed in the growth medium (control) in the absence of bacteria (Figure 2B). The hydrolysis level was significantly higher in the control compared to that observed in the Gram-positive cultures and significantly lower than the observed levels in the Gram-negative cultures (*p* value = 0.0001). Notably, the final pH of the cultures differed significantly compared to the growth media, and among the Gram-negative and Gram-positive strains tested. Moreover, we confirmed the presence of the *yjfP* esterase gene by PCR in one of the hydrolyzing strains, *E. coli* BW25113, as well as in one of the non-hydrolyzing strains, *E. faecium* W54 (Figure S3). 

The results from the control and presence of *yjfP* esterase gene in a strain that hydrolyzed the MPH and another strain that did not hydrolyze the MPH led us to suspect the role of bacterial esterases in the observed MPH hydrolysis. To confirm our hypothesis, we employed an *E. coli* BW25113 with a deletion of the *yjfP* gene encoding the esterase enzyme, *E. coli* BW25113^ΔyjfP^. Interestingly, the lack of the *yjfP* esterase did not result in any significant difference in the hydrolysis of MPH compared to the wild-type strain (*p* value = 0.1099) (Figure 2B).

Together with the spontaneous hydrolysis observed in culture medium (pH 7.0), these results hinted towards a possible effect of pH shifts in the MPH hydrolysis.

### 2.3. pH Shifts during Bacterial Growth Cause Hydrolysis of Methylphenidate in Bacterial Pure Cultures (An)aerobically

The spontaneous hydrolysis of MPH observed in bacterial growth medium in the absence of bacteria (Figure 2B) led us to investigate the role of pH in MPH hydrolysis. In bacterial cultures, where MPH was not metabolized, the pH measured after 24 h ranged between 4.0 and 5.5 (Table 1). In contrast, bacterial cultures that showed high levels of MPH hydrolysis had a pH between 7.5 and 8.0 (Table 1). Moreover, the *E. coli* BW25113 cultures had a slightly higher average pH of 7.8 compared to *E. coli* DSM1058 and *E. coli* DSM12250, where the average pH was 7.6; this was accompanied by a smaller percentage of MPH hydrolysis, 70% versus 50%, respectively (Figure 2B, Table 1). Indeed, Pearson r correlation analyses showed a strong positive correlation (r = 0.89, r^2^ = 0.79, *p* value = 0.0006) between MPH-hydrolyzing bacterial cultures and pH of the bacterial cultures after 24 h. These findings strongly suggest that the pH of the bacterial culture, and not bacterial metabolic activity, is responsible for the hydrolysis of MPH into its inactive form, RA. 

**Figure 3:**
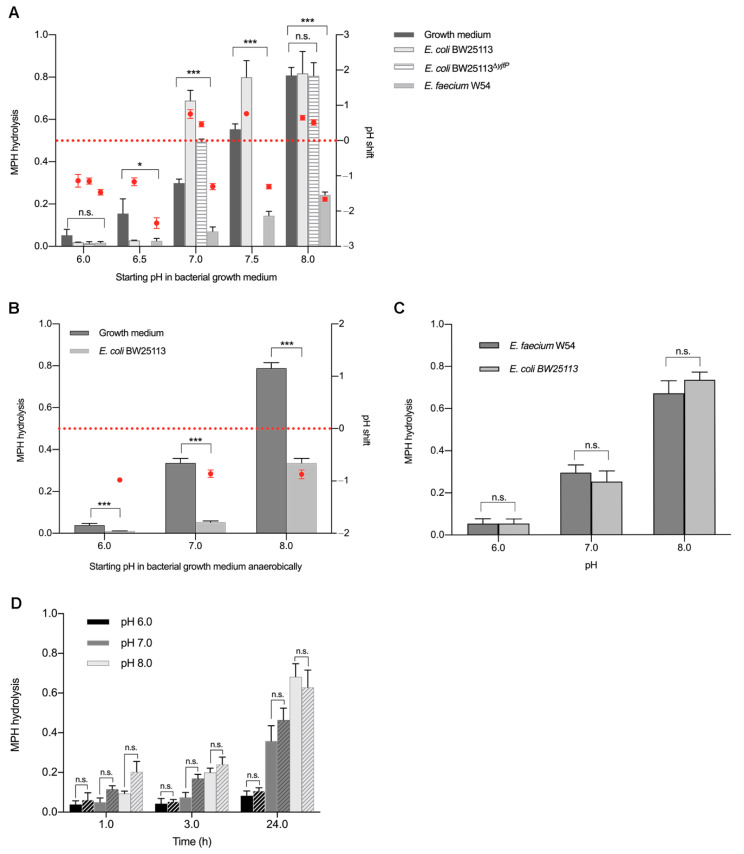
Effect of pH on methylphenidate hydrolysis in the presence and absence of bacterial esterases. Methylphenidate hydrolysis at different starting pH in (**A**) bacterial growth medium, *E. coli* BW25113, *E. coli* BW25113*^ΔyjfP^* and *E. faecium* W54 pure cultures, (**B**) *E. coli* BW25113 pure cultures anaerobically, (**C**) *E. coli* BW25113 and *E. faecium* W54 bacterial supernatants and (**D**) *E. coli* BW25113 cell lysates (solid bars) and phosphate buffer control (striped bars). Methylphenidate hydrolysis quantified in ng/µL and normalized to d10-ritalinic acid (left *y*-axis; grey bars) and pH changes during bacterial growth are shown as pH shift after 24 h (red dots; right *y*-axis). Error bars represent SD (*n* = 3). * *p* < 0.05, *** *p* < 0.001.

To determine whether gut bacterial metabolic activity plays any role in the observed hydrolysis of MPH, MPH stability was tested in the bacterial growth medium. Enriched beef broth was prepared at different pH values, ranging from 6.0 to 8.0 to resemble the pH values previously measured in the different bacterial cultures (Table 1), and was incubated aerobically with 50 µM MPH for 24 h. MPH hydrolysis was analyzed by HPLC-MS/MS. At pH 6.0, which resembles the pH measured in bacterial cultures that did not hydrolyze MPH, 80% of MPH remained intact, while 80% of the drug was hydrolyzed to RA in the control medium, without any bacteria at pH 8.0 (Figure 3A). 

To further confirm the observed association between MPH hydrolysis in bacterial cultures and the pH shift, we selected *E. coli* BW25113, which showed 70% hydrolysis of MPH into RA, and *E. faecium* W54, which did not hydrolyze the drug (Figure 2B), both strains harboring the yjfP esterase gene (Figure S3). *E. coli* BW25113 and *E. faecium* W54 were grown at pH values ranging from 6.0 to 8.0 at the start of incubation. Incubation was performed aerobically with 50 µM MPH for 24 h and MPH hydrolysis was analyzed by HPLC-MS/MS. Changes in pH after 24 h of incubation were measured and compared to the initial pH values (Table 2).

When *E. coli* BW25113 was grown in enriched beef broth at pH ≤ 6.5, a negligible amount of MPH was hydrolyzed to RA after 24 h of incubation with 50 µM MPH and the pH of the 24 h culture dropped to 5.0–5.5 (Figure 3A, Table 2). In contrast, when *E. coli* BW25113 was grown in enriched beef broth at pH ≥ 7.0 (the same pH of the culture plotted in Figure 2B), the pH of the culture rose to 7.5–8.5 after 24 h of incubation with MPH and 70–90% of MPH was hydrolyzed to RA (Figure 3A, Table 2). On the other hand, when *E. faecium* W54 was grown in enriched beef broth at pH ≤ 6.5, the pH of the culture dropped below 5.0 after 24 h of incubation with MPH and a negligible amount of MPH was hydrolyzed to RA. When *E. faecium* W54 was grown at pH ≥ 7.0, the pH of the cultures dropped to values between 6.5 and 5.5 and this was accompanied by only 20% MPH hydrolysis (Figure 3A, Table 2). As the statistical analysis revealed, acidification or alkalization of the media produced significative differences between bacterial cultures and their corresponding pH medium controls (Table 2). These data show that it is pH rather than bacterial esterases that is responsible for the MPH hydrolysis. To further confirm our conclusion, the esterase mutant *E. coli* BW25113^ΔyjfP^ (Figure S3) was also incubated with MPH at different pH levels. MPH hydrolysis was studied in this case at the pH values that had been observed to have the biggest effect on MPH stability: 6.0, 7.0, and 8.0 (Figure 3A). Knocking out the esterase did not result in a significant difference with the wild-type (*p* value = 0.3796). Taken together, the results indicate that the MPH hydrolysis by gut bacteria observed in Figure 2A,B was likely to be due to pH shifts over the duration of bacterial incubation with MPH.

To exclude the possibility that MPH hydrolysis is affected by the presence of oxygen in bacterial growth media, *E. coli* BW25113 was grown anaerobically and incubated with 50 µM MPH for 24 h at different starting pH. Growth medium was used as a control. Similar to its hydrolysis under aerobic conditions, MPH hydrolysis to RA was observed in both bacterial growth medium controls and *E. coli* BW25113 under anaerobic conditions in a pH-dependent manner (Figure 3B). However, in *E. coli* BW25113 anaerobic cultures, pH changes during incubation were different compared to those observed under aerobic conditions. Under anaerobic culturing, acidification of the bacterial culture took place. Cultures with a starting pH 6.0 and 7.0 showed a drop in pH to around 5.0 and 6.0, respectively, after 24 h, and accordingly, a neglectable hydrolysis of MPH. When the cultures had a starting pH of 8.0, acidification to pH 7.0 lead to around 30% of MPH hydrolysis (Figure 3B, Table 2). Collectively, these data indicate that the pH shift derived from changes in bacterial metabolism under anaerobic conditions is responsible for the MPH hydrolysis.

To exclude the possibility that certain components within the bacterial growth media could be catalyzing the hydrolysis of MPH, *E. coli* BW25113 and *E. faecium* W54 cultures were grown to late exponential phase to reach high bacterial density (Figure S4) and the supernatants were collected, filtered, and incubated with 50 µM MPH. pH values of *E. faecium* W54 supernatants, which were around 5.5, were adjusted to 6.0, 7.0 and 8.0, respectively, to resemble the pH previously measured in the different bacterial cultures (Figure 2C). Interestingly, incubation of MPH with *E. faecium W54* supernatants at pH 6 resulted in 10% hydrolysis of MPH to RA, but levels of hydrolysis increased with increased pH levels: 20–30% at pH 7.0 and 60–70% at pH 8.0, respectively (Figure 3C), in a similar manner as previously observed (Figure 3A). When *E. coli* BW25113 supernatants were adjusted to the same pH values, no significant difference in MPH hydrolysis was observed when compared to the *E. faecium* W54 supernatants (*p* value = 0.34) (Figure 3C). 

Finally, to exclude the possibility that intracellular bacterial esterases could be contributing to the hydrolysis of MPH, cell lysates of *E. coli* BW25113, known to carry the predicted *yjfP* esterase gene (Figure S3), were incubated with MPH at pH 6.0, 7.0 and 8.0, respectively, to resemble the pH previously measured in the different bacterial cultures (Figure 2B). Samples were taken at different timepoints to compare the rate of hydrolysis in lysates and buffer controls. MPH hydrolysis in *E. coli* BW25113 lysates happened in the same way as in the bacterial cultures. At acidic pH 6.0, only around 10% of MPH was hydrolyzed and the hydrolysis increased to 30–40% at pH 7.0 after 24 h. When the cell lysates were incubated with MPH at alkaline pH 8.0, around 70% of the drug was converted to RA (Figure 3D). The same was observed in the phosphate buffer controls, where no significant differences were observed after 24 h when compared to the cell lysates (*p* value = 0.72) (Figure 3D). At 1 and 3 h of incubation, no significant difference between cell lysates and buffer controls was found (*p* value = 0.11, 0.18, respectively). Collectively, our data clearly indicate that the hydrolysis of MPH observed in our experiments resulted from pH-dependent spontaneous non-enzymatic conversion rather than from direct bacterial metabolization.

## 3. Discussion

Our in vitro studies identified pH shifts as a major factor affecting the stability of the main medication for ADHD, MPH, while gut bacterial metabolization did not seem to contribute to the MPH hydrolysis. Our results are in agreement with a recent study that investigated a wide variety of drugs for their possible degradation by colonic gut bacteria. MPH was among the drugs that were metabolized the least; only around 20% of MPH was hydrolyzed at pH ≤ 6 [27]. pH was not considered as a factor for non-enzymatic hydrolysis of the drug and metabolism of the drug was only studied in colonic bacteria, which require anaerobic conditions. Nonetheless, we focused our studies on small intestinal bacteria, given that the majority of MPH is absorbed before it reaches the colon [12].

Although our initial experiments using small intestinal luminal content from rats (Figure 2A) suggested the capability of gut microbiota to metabolize MPH, our further observations of the spontaneous hydrolysis of MPH under physiological conditions (37 °C, pH 7.0) (Figure 2B), as well as the results from incubation of MPH in growth media in the presence and absence of bacterial esterases, uncovered a pH-dependent MPH hydrolysis, irrespective of the presence of bacteria (Figure 3). The complex bacterial community present in the luminal content could have caused fluctuations in the pH levels during the 24 h period of incubation with MPH. Thus, we anticipate that the MPH hydrolysis observed when incubated with the small intestinal luminal content was mainly caused by an elevation of pH levels during the course of incubation. 

To our knowledge, this is the first report that describes the effect of pH on the stability of MPH in aerobic bacterial cultures at 37 °C under the different pH levels found in the small intestine. Besides the analytical profile of MPH, where significant basic degradation was observed only at extreme temperatures (100 °C) [28], the stability of MPH at different pH levels has not been thoroughly investigated, or at least, this information is not readily available. Although detailed pharmacokinetics and pharmacodynamics for the MPH have been described [29], no further information could be found regarding non-enzymatic hydrolysis in biologically relevant conditions that to some extent mimic the gastrointestinal tract. A study of drug–excipient interactions performed forced degradation experiments and identified RA as the main degradation product of MPH [30]. In that study, acid degradation in 1N HCl solution at 80 °C for 2 h resulted in 15% of MPH hydrolysis. While base degradation in 1N NaOH solution at room temperature for 0.3 h resulted in as much as 33% of MPH hydrolysis. Another recent study also performed forced degradation experiments of MPH in 1N HCl and 1N NaOH solutions at 60 °C during 30 min. In line with our observations, significant MPH hydrolysis was only observed in alkaline conditions [31]. Moreover, hydrolysis of MPH was also reported in static water, where MPH was completely hydrolyzed to RA within 37 h at 20 °C [32]. Lastly, hydrolysis in phosphate buffer (pH 7.4) at 37 °C was also observed in a different study, with 40% of MPH being hydrolyzed after 8 h [33]. Although these studies indicate that MPH can be spontaneously hydrolyzed to RA, none of the studies accounted for the physiological conditions of the small intestine that were considered in our study. The temperature, pH range and the complex luminal composition of the gut used here mimic, to a certain extent, the environment to which MPH is exposed and which could very likely lead to non-enzymatic hydrolysis of the drug before its absorption in the small intestine. 

Our data suggest that changes in the pH along the gastrointestinal tract may induce non-enzymatic degradation of the MPH and therefore account, at least in part, for the low bioavailability of the drug. In the small intestine, luminal pH varies from 6.0 in the more proximal regions to 8.0 when reaching the ileum [34]. Moreover, these levels are highly susceptible to vary based on the dietary composition. According to our data, this pH increase along the small intestine would result in 60% of MPH hydrolysis by the time it reaches the ileum (Figure 3A). Preventing MPH from reaching an increased pH in the ileum by taking the medication under fasting conditions could improve its bioavailability, as MPH would be absorbed higher up in the small intestine [12], where a pH below 7 should limit the non-enzymatic hydrolysis to around 10% (Figure 3A). Reports on the pharmacokinetics of MPH comparing fed and fasting conditions are scarce and reveal contradictory results [35,36]. 

The gut microbial composition and its metabolization products from the interaction with different food components is another key factor that can influence the small intestinal pH [37] and cause interindividual differences in MPH bioavailability. For example, pH measured in ileostomy effluent from an ileostomy patient raised from 5.6 in the morning to 6.8 in the afternoon due to changes in feeding cycles [37] indicating that pH changes can indeed take place in the small intestine due to bacterial metabolism. Moreover, protein and amino acid deamination by gut bacterial metabolization results in the production of amine groups and ammonia that can also increase luminal pH [38]. In rodents, for example, a high fiber diet led to an increase in small intestinal pH from 7.5 to 8.0 [19]. Luminal pH changes derived from bacterial metabolization of dietary components could lead to 30% of MPH spontaneous hydrolysis as soon as luminal pH reaches 7.0, and up to 60% of the drug would be degraded if spontaneous hydrolysis of MPH occurred prior to its absorption (Figure 3). Thus, interindividual differences in small intestinal microbial composition and/or diet could be a critical factor in MPH presystemic hydrolysis by shifting luminal pH either towards acidic pH, providing stability for MPH, or alkaline pH, which would prompt MPH hydrolysis. 

Collectively, the present study shows that MPH is subjected to spontaneous hydrolysis in a pH-dependent manner in bacterial cultures grown in complex media that resemble the gut lumen and that gut bacteria are not likely to directly influence the drug bioavailability though their metabolization. However, an indirect effect of the gut microbiota through the production of pH-modifying metabolites could not be ruled out. Our findings provide a significant addition to previous studies reporting on the low bioavailability and interindividual variation in the response to MPH [7,8]. However, the main limitation of the current study is the lack of clinical measurements in ADHD patients. It is crucial to assess whether the interindividual variation in response to MPH is related to differences among subjects in their small intestinal intraluminal pH caused by differences in diet, gut microbiota composition and their metabolic products, administration of other medications including antacids, MPH formulation administered, and whether the drug is taken in fasting or fed conditions. To this end, ex vivo human studies, via the application of gastrointestinal sampling capsules [39], for example, aiming at measuring small intestinal pH in response to different dietary components and medication are needed. 

## 4. Material and Methods

### 4.1. Bioinformatics

Protein sequences of human CES1 (NCBI accession: AAI_10339.1), *E. coli* yjfP (NCBI accession: ANK_04958.1) and *B. subtilis* pnbA (NCBI accession: KIX_83209.1) were retrieved from the NCBI database and a BLAST search was locally carried out against the translated HMP Reference Genome (HMRGD) sequence database, which was filtered to contain only the GI tract as the body site. Blastp was run in default configuration, and hits with less than 90% query cover or less than 30% identity were discarded to still stay in the safe space for homology modelling. From the resulting sequences, orthologues were inferred by running OrthoFinder with the-M option set to “msa”. The 15 closest (with highest identity) orthologues to each of the initial seed sequences were extracted, and a multiple sequence alignment was performed using MUSCLE in default mode. The alignment served as an input for RAxML for phylogenetic tree inference using PROTGAMMAAUTO as the model and performing rapid bootstrap analysis with 100 cycles as well as best-scoring ML tree search. This yielded a GAMM + WAG + F model with empirical base frequencies. The unfiltered results of the default BLAST search for yjfP orthologues were also included in this study to broaden our search of gut bacteria harboring esterases below the 30% identity cutoff (Figure S1). The resulting trees were further visualized with iTOL.

### 4.2. Rat Luminal Content

The luminal small intestinal content of wild-type Groningen (WTG) rats (*n* = 5) was collected in sterile Eppendorf tubes by gentle pressing along the entire cecum and was snap frozen in liquid N_2_ and stored at −80 °C. Ten percent (*w/v*) suspensions of the luminal content were grown in enriched beef broth based on SHIME medium (Table S1) [25]. Bacterial cultures within the inoculum were allowed to grow for 3 h, followed by supplementation with 50 µM methylphenidate (MPH) hydrochloride tablets (10 mg, Mylan; provided by Dr. R. Pereira; Medical Center Kinderplein, Rotterdam, the Netherlands). MPH and were incubated at 37 °C in aerobic conditions with shaking at 220 rpm. Samples were collected at 0 and 24 h for HPLC-MS/MS analysis.

### 4.3. Pure Bacterial Cultures

*L. salivarius* W1, *L. plantarum* W24 and *E. faecium* W54 strains were obtained from Winclove Probiotic B.V. *C. ammoniagenes* DSM20306, *E. coli* DSM1058 and *E. coli* DSM12250 were obtained from the German Collection of Microorganisms and Cell Cultures (DSMZ). Additionally, the lab strains *E. coli* BW25113 [40] and the vancomycin-resistant strain *E. faecalis* V583 [41] were used in this study. All bacterial strains were grown in incubators (New Brunswick Scientific) at 37 °C aerobically in enriched beef broth (Table S1). For experiments where the effect of pH on MPH hydrolysis was studied (Figure 3), culture media were prepared at different pH values. To do so, buffer solutions of KH_2_PO_4_/K_2_HPO_4_ were prepared at different concentrations to obtain the desired pH when adding them to the media. Strains that required shaking for proper growth (all *E. coli* strains, *C. ammoniagenes* DSM20306, *L. salivarius* W24 and *L. plantarum* W1) were grown with continuous agitation at 220 rpm. *E. coli* BW25113 was also grown anaerobically (10% H_2_, 10% CO_2_, 80% N_2_) in a Don Whitley Scientific DG250 Workstation (LA Biosystems, Waalwijk, the Netherlands) at 37 °C and without agitation. Bacteria were inoculated from −80 °C glycerol stocks and grown overnight. Before the experiment, cultures were diluted to 1% in fresh enriched beef broth medium and were grown until late exponential phase to reach high bacterial density (Figure S4). Growth was followed by measuring optical density (OD) at 600 nm in a spectrophotometer. A measure of 50 µM MPH was added to the cultures and samples were taken at 0 and 24 h for HPLC-MS/MS analysis.

### 4.4. Amplification of yjfP Esterase by PCR

The presence of the predicted *yjfP* esterase gene was verified in *E. coli* BW25113, *E. coli* BW25113^Δ*yjfp*^ and *E. faecium* W54 gDNA. yjfP from *E. coli* (accession CP15085) was amplified by PCR using the MyCycle thermal cycler (Bio-rad, California, United States) and the following primers: forward (5′-ATGATTGAAATAGAATCACGCGAGCTG-3′) and reverse (5′-TTAAAGATGCTGGCG-GAAAAATGTCAC-3′) were used to generate a product of 750 bp.

### 4.5. HPLC-MS/MS Sample Preparation and Analysis

In order to monitor the levels of MPH and ritalinic acid (RA) hydrochloride solution (1 mg/mL as a free base, Sigma-Aldrich, Amsterdam, the Netherlands) in growth media and bacterial cultures, samples were collected by adding 100 µL of culture to 400 µL of 100% methanol. The internal standard d10-ritalinic acid (d10-RA) hydrochloride solution (100 µg/mL as a free base; Sigma-Aldrich, the Netherlands) was added to all samples at a final concentration of 2 ng/µL as an internal standard for accurate quantification. Samples were then centrifuged at 14,000 rpm for 15 min at 4 °C. Supernatants were transferred to a clean tube and methanol was evaporated using a Savant speed-vacuum dryer (SPD131, Fisher Scientific, Landsmeer, the Netherlands). Finally, samples were reconstituted in 500 µL of water. 

Sample analysis was performed using a Shimazu HPLC system consisting of an SIL-20AC autosampler, a CTO-20AC column oven and LC-20AD liquid chromatograph pumps. Chromatography separation was achieved using a Waters CORTECS C18+ column (100 × 2.1 mm; 2.7 µm). The mobile phase consisted of a mixture of water (A) and acetonitrile (B) both containing 0.1% formic acid. A flow rate of 0.25 mL/min was used with a linear gradient: 5% (B) for 5 min, followed by an increase to 80% (B) in 5 min, which was kept for 3 min to wash the column and then re-turned to initial conditions for 2 min. The HPLC was coupled to an API3000 triple-quadrupole mass spectrometer (Applied Biosystems/MDS Sciex) via a turbo ion spray ionization source. Ionization was performed by electrospray in positive mode and selected reaction monitoring (SRM) was used to detect the metabolites. The SRM transitions were: m/z 234 to 84 for MPH, 220 to 84 for RA and 230 to 84 for d10-RA. Other parameters were set as follows for all transitions: declustering potential 15 V, entrance potential 7 V, focusing potential 65 V, collision energy 30 V and collision cell exit potential 14 V.

### 4.6. Calibration Standards and Biological Matrices

MPH standard was obtained by extraction from MPH hydrochloride tablets (10 mg) as follows: one tablet was crushed in a mortar and the resulting powder was diluted in 10 mL of a mixture containing acetonitrile, methanol, and acetate buffer pH 4 (0.2 M CH_3_COONa) in a ratio of 30:50:20, respectively. The solution was mixed with a magnetic stirrer for 10 min and was allowed to stand until the solid phase containing the insoluble components of the tablets had precipitated. Next, the polar liquid phase containing MPH was collected with a syringe and sterilized using 0.2 µm filters. This resulted in stock solutions of 1 mg/mL of MPH, which were stored at −20 °C until further use.

For MS quantification, calibration curves were obtained in different matrices to account for matrix effects in the detection of MPH and RA. Calibration samples containing MPH and RA in a concentration range of 0.01 to 5 ng/µL were prepared in methanol and 2 ng/µL of d10-RA was added as an internal standard to correct for intrasample variation of MPH and RA. Next, methanol was removed by vacuum centrifugation and samples were reconstituted in 500 µL of the relevant biological matrix. Two types of biological matrices were prepared. For quantification in pure bacterial cultures, *E. coli* BW25113 cultures were grown to late exponential phase (Figure S4), cells were removed by centrifugation at 14,000 rpm for 15 min and the supernatants were filtered and used for reconstitution. Similarly, a pool was made combining the small intestinal content of 5 rats used in Figure 2A to obtain a complex matrix for this experiment. The pooled inoculum was allowed to grow for 3 h and supernatant was obtained by centrifugation and filtering as explained before to be used for reconstitution of the calibration curves. Linearity of the detection of MPH and RA in both biological matrices is shown in Figure S2.

### 4.7. Cell Lysate Preparation and Assay

Overnight cultures of *E. coli* BW25113 were harvested and pellets were washed 3 times in 1 mL 1X PBS. After washing, pellets were resuspended in 1 mL of ice-cold PBS. Before lysis, 2 μg/mL DNase and 200 μg/mL lysozyme were added to each solution and incubated at 37 °C for 15 min. Next, sonication was performed for 2 min in order to lyse the cells. Twelve cycles were applied consisting of 5 s of sonication and 5 s of cooldown each cycle at an amplitude of 6.0. After lysis, clear cell lysates were obtained by centrifugation for 20 min at 14,000 rpm at 4 °C. Total protein concentration was determined by Bradford assay. Cell lysates assays were performed at 37 °C with 50 µM MPH. Incubations were performed at 0.2 mg/mL of cell lysates in phosphate buffer at pH 6.0, 7.0 and 8.0. Samples were collected and quenched in 400 μL ice-cold 100% methanol at 0, 1, 3 and 24 h, respectively. Samples were stored at −20 °C until sample preparation and analysis by HPLC-MS/MS.

### 4.8. Statistical Analysis

All statistical tests and linear regression models were performed using GraphPad Prism 7.

## 5. Conclusions

The present study shows, for the first time, that MPH is hydrolyzed in a non-enzymatic pH-dependent manner in physiologically relevant conditions resembling the small intestinal environment in which this drug is absorbed. Our observations suggest that bioavailability of MPH is unlikely to be influenced by bacterial esterases, but rather intraluminal pH differences of ADHD patients. Further clinical studies are required to verify, in humans, whether intestinal pH has an effect on the bioavailability of MPH, and whether this can be corrected through dietary intervention. 

## Figures and Tables

**Table 1:** pH measurements in pure and complex bacterial cultures. Summary table of pH values measured in pure and complex bacterial cultures starting at pH 7.0 after 24 h of incubation with 50 μM methylphenidate.

Pure Cultures	Starting pH	pH after 24 h	S.D.	Complex Cultures	Starting pH	pH after 24 h	S.D.
Growth medium	7.00	7.02	0.01	Rat 1	7.00	7.24	N.A.
*P. fluorescens* MFY63	7.00	8.01	0.15	Rat 2	7.00	7.62	N.A.
*E. coli* BW25113	7.00	7.80	0.07	Rat 3	7.00	7.85	N.A.
*E. coli* BW25113*^ΔyjfP^*	7.00	7.54	0.05	Rat 4	7.00	8.52	N.A.
*E. coli* DSM12250	7.00	7.60	0.05	Rat 5	7.00	6.68	N.A.
*E. coli* DSM1058	7.00	7.64	0.04				
*E. faecalis* V583	7.00	5.40	0.14				
*E. faecium* W54	7.00	5.73	0.08				
*L. salivarius* W24	7.00	4.07	0.05				
*L. plantarum* W1	7.00	4.19	0.13				

**Table 2:** pH measurements in pure bacterial cultures. Summary table of pH values measured in pure bacterial cultures after 24 h of incubation with 50 μM methylphenidate at different starting pH values. Standard deviation (S.D.) represents 3 biological replicates. Unpaired *t*-test was performed to compare methylphenidate hydrolysis in bacterial cultures to the corresponding pH controls in bacterial growth medium.

Starting pH	*E. coli* BW25113			*E. faecium* W54			*E. coli* BW25113^Δ^*^yjfP^*			*E. coli* BW25113 (anaerobic)		
	pH 24 h	S.D.	*p*	pH 24 h	S.D.	*p*	pH 24 h	S.D.	*p*	pH 24 h	S.D.	*p*
6.0	4.86	0.18	0.1098	4.20	0.43	0.1013	4.85	0.08	0.1797	5.02	0.12	0.0066
6.5	5.33	0.11	0.0339	4.30	0.26	0.0339	N.A.	N.A.	N.A.	N.A.	N.A.	N.A.
7.0	7.63	0.07	0.0002	5.70	0.08	0.0001	7.46	0.07	0.0001	6.14	0.07	0.0003
7.5	8.26	0.06	0.0066	6.22	0.11	0.00002	N.A.	N.A.	N.A.	N.A.	N.A.	N.A.
8.0	8.64	0.06	0.8851	6.40	0.16	0.00001	8.51	0.07	0.9774	7.13	0.08	0.0002

## Data Availability

Data is contained within the article or Supplementary Material.

## Supplementary Materials

The following are available online at https://www.mdpi.com/article/10.3390/ph14080733/s1, Figure S1: In-silico analysis of bacterial homologues of *E. coli* yjfP, Table S1: Constituents of enriched beef broth medium used in this study, Figure S2: Calibration curves obtained in the two different biological matrices used in this study, Figure S3: PCR of bacterial esterases, Figure S4: Growth curves of the strains used in this study.
