# Supplementary material for: Stability of Methylphenidate under Various pH Conditions in the Presence or Absence of Gut Microbiota
Source: Pharmaceuticals (Basel). 2021 Jul 27;14(8):733. doi: 10.3390/ph14080733 (PMC8398889; doi:10.3390/ph14080733)
Supplement: Supplementary file 1 [file pharmaceuticals-14-00733-s001.zip › pharmaceuticals-1271791-supplementary.pdf]

Supplementary figures

Table S1. Constituents of enriched beef broth medium used in this study.

Salt and carbon sources		Vitamin solution		Trace elements	
Component	g/L	Component	g/L	Component	g/L
Glucose	2.000	D-Biotin	0.0020	EDTA	1.000
NaCl	0.080	D-Panthoteic acid	0.0100	$ZnSO_4.7H_2O$	0.178
K_2HPO_4	5.310	Ca2.Nicatinamide	0.0050	$MnSO_4.7H_2O$	0.452
KH_2PO_4	2.650	Vitamine B12	0.0005	$FeSO_4.7H_2O$	0.100
$NaHCO_3$	0.400	Para-aminobenzoic acid	0.0050	$CoSO_4.7H_2O$	0.181
Beef extract	5.000	Riboflavin	0.0050	$CuSO_4.7H_2O$	0.010
Yeast extract	3.000	Folic acid	0.0020	H_3BO_3	0.010
Peptone	0.600	Pyridoxal-5-Phosphate	0.0100	$Na_2MoO_4.2H_2O$	0.010
$CaCl_2$	0.008	Vitamin K1	0.0005	$NiSO_4.6H_2O$	0.111
$MgSO_4$	0.008	Thiamin HCl	0.0040		
Cysteine	0.500				

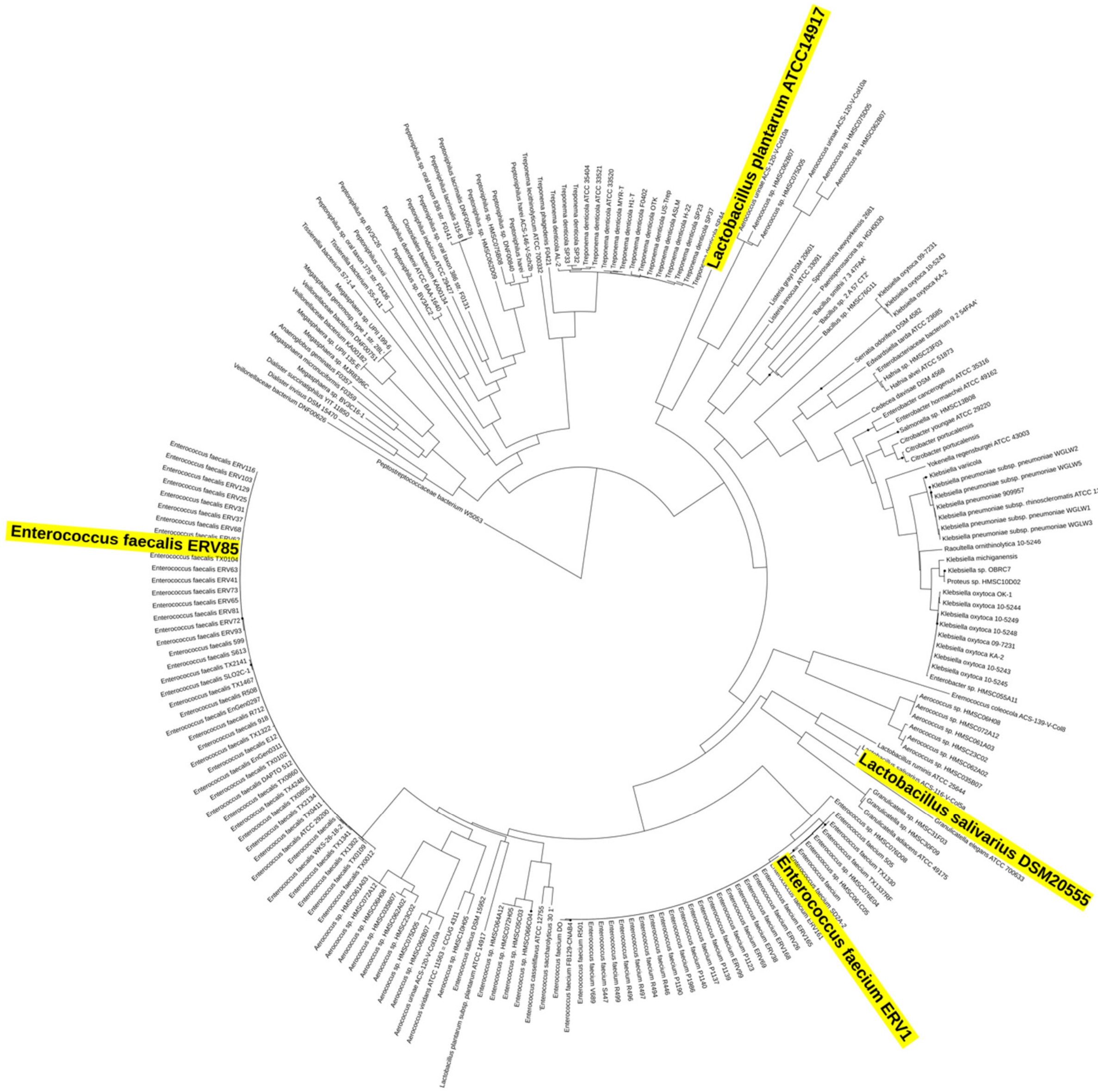

Figure S1. ***In-silico*** **analysis of bacterial homologues of *E.coli* yjfP.** Phylogenetic tree created with iTOL online tool showing gut bacterial strains carrying yjfP esterase orthologues below the 30% identity cut-off of the alignment model. Highlighted in yellow are the bacteria selected for our screening of methylphenidate hydrolyzing bacteria.

A

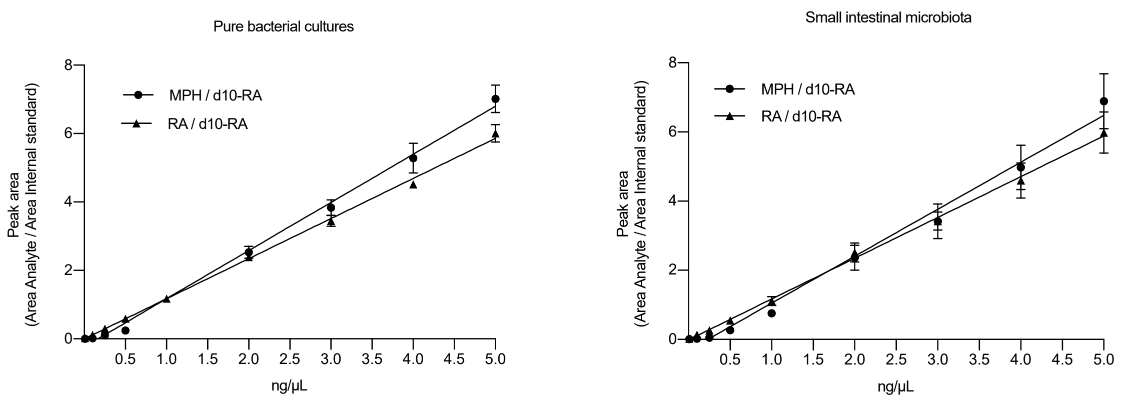

C

Matrix	Calibration line slope	Calibration line intercept	Correlation coefficient (r^2)
Bacterial pure cultures			
MPH	1.407 ± 0.03511	-0.2332 ± 0.09148	0.9983
RA	1.172 ± 0.01751	-0.00112 ± 0.04342	0.9984
Small intestinal microbiota			
MPH	1.398 ± 0.04149	-0.3152 ± 0.1029	0.9784
RA	1.152 ± 0.02431	-0.01052 ± 0.06028	0.9890

Figure S2. (A, B) Calibration curves obtained in the two different biological matrices used in this study: **(A)** pure bacterial cultures of *E. coli* BW25113 and **(B)** pool of small intestinal content of 5 WTG rats. Peak areas of methylphenidate (MPH) and ritalinic acid (RA) are normalized to the peak area of the internal standard d10-Ritalinic acid (d10-RA). **(C)** Linearity of the calibration curves fitted with a linear regression model. Data represents 3 biological replicates and error bars represent standard deviation.

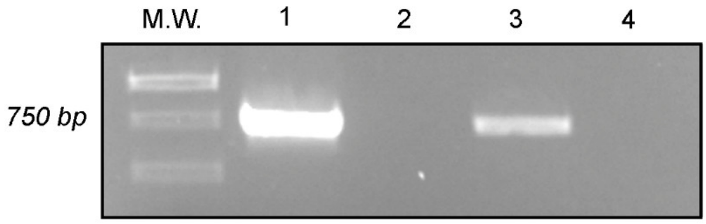

Figure. S3 PCR of bacterial esterases. Amplification of the *E. coli* yjgP gene (accession: CP015085) in **(1)** *E. coli* BW25113, **(2)** *E. coli* BW25113$^{\Delta yjfP}$, **(3)** *E. faecium* W54 and (4) no template control.

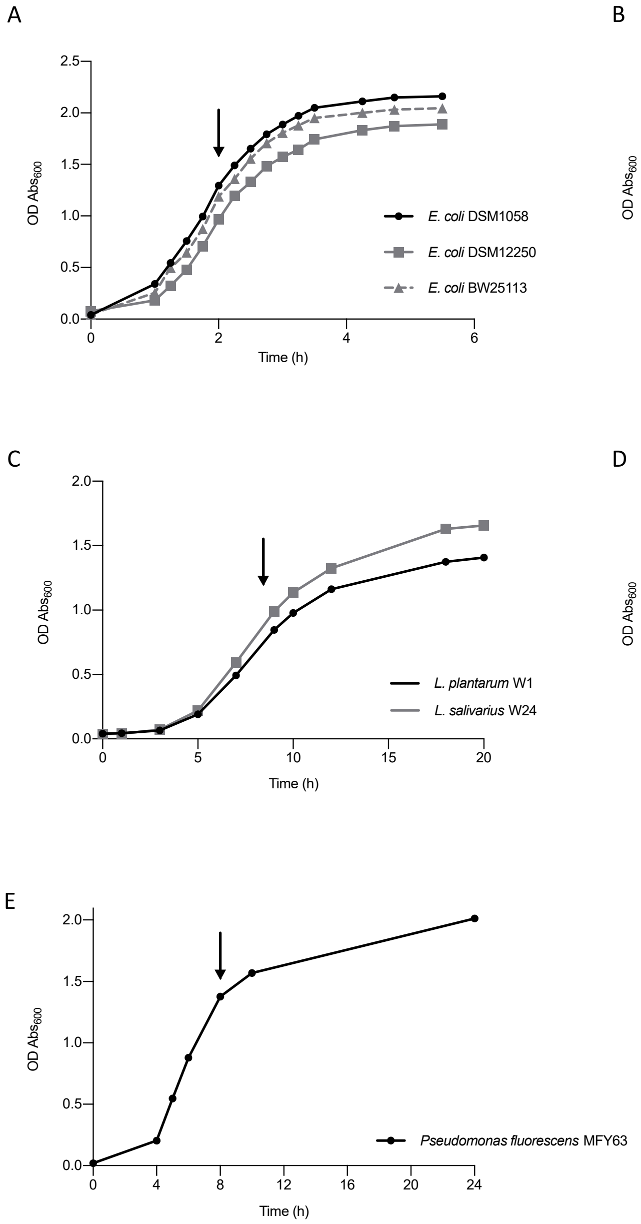

Figure S4. Growth curves of the strains used in this study. Optical density measured as the absorbance at 600 nm is plotted over time in aerobic cultures of (A) *E. coli* strains (BW25113, DSM11250 and DSM1058) grown at 37°C, 220 rpm; (B) *Enterococcus* strains (*E. faecalis* V583 and *E. faecium* W54) strains grown at 37°C without agitation (C) *Lactobacillus* strains (*L. plantarum* W1 and *L. salivarius* W24) grown at 37°C, 220 rpm *Enterococcus* strains (*E. faecalis* V583 and *E. faecium* W54) strains grown at 37°C without agitation; **(D)** *C. ammoniagenes* DSM20306 grown at 37°C, 220 rpm and **(E)** *P. fluorescens* MFY63 grown at 37°C, 220 rpm. Arrows indicate the late exponential phase when MPH was added.
